# ID 291 - Aproximando a ATS da Comunidade: iniciativas locais de divulgação junto aos usuários de um hospital no Pará

**DOI:** 10.5327/2237-9622.2025.v34s1.291

**Published:** 2025-11-25

**Authors:** Cristina Maria Maués da Costa, Daniele Rodrigues Assunção, Lilian Pereira da Silva Costa, Valéria Oliveira da Trindade

## Abstract

**Introdução:**

Este trabalho é fruto do projeto de extensão "Empoderamento dos pacientes na perspectiva da Avaliação de novas Tecnologias em Saúde no SUS", financiado pela Pró-Reitoria de Extensão da Universidade Federal do Pará (UFPA). Teve como finalidade principal a divulgação aos usuários do Hospital Universitário João de Barros Barreto (HUJBB) – profissionais de saúde, pacientes e estudantes – da Avaliação de Tecnologias em Saúde (ATS) e sobre como participar efetivamente do processo de incorporação dos medicamentos para uma determinada doença por meio das consultas e chamadas públicas da Comissão Nacional de Incorporação de Tecnologias em Saúde (Conitec). O projeto desenvolveu ações educativas para divulgar e estimular a participação social dos usuários do SUS mediante o compartilhamento de conhecimentos sobre o tema da ATS, em linguagem acessível a todos. Entre as estratégias adotadas, está a produção de alguns materiais educativos, feitos em formato de fôlder pela plataforma de edição Canva, tendo como referência as orientações da Conitec. Todos os fôlderes continham as seguintes informações básicas: título, com a informação da consulta pública vigente, passo a passo de como participar da consulta pública com imagem ilustrativa do formulário de contribuição, o papel da Conitec e a importância e o objetivo da consulta ou chamada pública, e a seguir um resumo da estrutura da pergunta PICO. Nesse contexto, foram realizadas palestras, com o objetivo de disseminar as consultas públicas vigentes, como a consulta pública sobre a atualização do Protocolo Clínico e Diretrizes Terapêuticas (PCDT) da Retinopatia Diabética, sobre a incorporação do medicamento elexacaftor associado a tezacaftor e ivacaftor para o tratamento dos pacientes com fibrose cística, as chamadas públicas para os pacientes de dermatite atópica, entre outros, além de banner sobre o papel dos profissionais farmacêuticos na ATS. Foram distribuídos fôlderes impressos e divulgados entre os profissionais de saúde por meio eletrônico e redes sociais, realizando uma pequena explicação e tirando dúvidas que surgiam. Essas palestras contaram com a ajuda dos profissionais do Núcleo de Avaliação de Tecnologias em Saúde do Complexo Hospitalar Universitário da UFPA (Nats/CHU-UFPA) na organização – em especial, da farmacêutica do setor do Programa do Componente Especializado da Assistência Farmacêutica e de duas acadêmicas do Curso de Farmácia da UFPA, vinculadas ao projeto de extensão. Essa participação ativa nas palestras e atividades educativas colaborou para a explicação dos processos de ATS e da importância da participação social nas consultas e chamadas públicas. Assim, essa atuação fortaleceu o projeto ao conectar os pacientes e profissionais de saúde sobre as informações necessárias para que pudessem contribuir de maneira mais efetiva e ativa no processo decisório, empoderando-os para participar diretamente na escolha de novas tecnologias e medicamentos incorporados ao Sistema Único de Saúde (SUS). Entre as perspectivas futuras, estão: disseminar esses informativos aos alunos da graduação em Farmácia por meio da Universidade Federal do Pará; além disso, a realização de palestras sobre vários temas da ATS, para divulgar os principais conceitos e fluxos de incorporação de tecnologias no SUS. Somando-se a isso, a criação de um perfil, na rede social Instagram para propagar os conhecimentos sobre a ATS e o papel da Conitec no SUS.

**Método:**

Roteiro da apresentação: 1) apresentação dos autores do trabalho; 2) introdução do tema e dos objetivos da iniciativa, descrição da metodologia e dos produtos obtidos; 3) conclusão e perspectivas futuras. Itens/equipamentos necessários para a execução: projetor de imagens conectado à internet.

